# The Preparation and Identification of a Monoclonal Antibody against Citrinin and the Development of Detection via Indirect Competitive ELISA

**DOI:** 10.3390/toxins9030110

**Published:** 2017-03-17

**Authors:** Shimuye Kalayu Yirga, Sumei Ling, Yanling Yang, Jun Yuan, Shihua Wang

**Affiliations:** Fujian Key Laboratory of Pathogenic Fungi and Mycotoxins, Key Laboratory of Biopesticide and Chemical Biology of The Education Minister, School of Life Science, Fujian Agriculture And Forestry University, Fuzhou 35002, China; kalayu.yirga@yahoo.com (S.K.Y.); Lsmpu2008@163.com (S.L.); yylwsh@sina.com (Y.Y.); yjmail2008@126.com (J.Y.)

**Keywords:** citrinin, conjugation, cell fusion, monoclonal antibody, ic-ELISA

## Abstract

Citrinin (CTN) is a hepato-nephrotoxic mycotoxin produced by fungi genera of *Aspergillus*, *Monauscus*, and *Penicillium.* CTN contaminates grains, fruits, juices and vegetables, and causes various toxic effects to humans and animals. It has small molecular weight, which is non-immunogenic to animals. Thus, CTN was conjugated to bovine serum albumin (BSA) and ovalbumin (OVA), respectively, by amide bonds using 1-ethyl-3-(3-dimethylaminopropyl) carbodiimide hydrochloride (EDC) and *N*-hydroxysuccinimide (NHS). Mice were immunized with CTN-BSA conjugates, and spleen cells of the immunized mice were fused with Sp2/0 myeloma cells to obtain 21H27 hybriodoma cell. Ascitic fluid of hybridoma cell was produced in mice abdomen, and purified using caprylic/ammonium sulfate precipitation method. The 21H27 anti-CTN mcAb was the IgG2a antibody subclass, and cross-reactivity results indicated that anti-CTN mcAb is specific to CTN with high affinity (2.0 × 10^8^ L/mol). Indirect competitive ELISA (ic-ELISA) results showed that the linear range of detection was 0.01–5.96 ng/mL and the IC_50_ was 0.28 ng/mL with a lower detection limit (LOD) of 0.01 ng/mL. The average recovery was 93.8% ± 1.6% with a coefficient variation of 1.0%–4.3%. Hence, anti-CTN mcAb secreted by 21H27 hybridoma cell was successfully produced and can be used to detect CTN contaminated feed and foodstuffs.

## 1. Introduction

**Figure 1:**
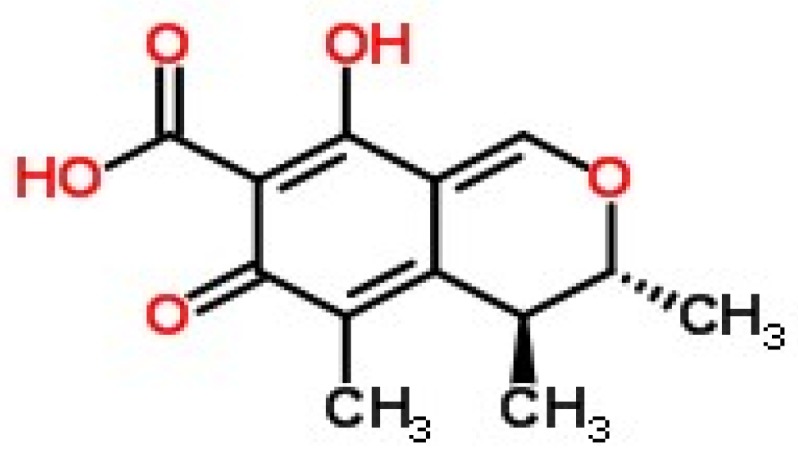
The structure of citrinin (CTN) [16].

Mycotoxins are poisonous chemical compounds produced by fungi which contaminate human food and animal feeds [1]. Citrinin (CTN) (Figure 1) has a molecular formula of C_13_H_14_O_5_ and is one of the oldest mycotoxins originally isolated from the secondary metabolites of *Penicillium citrinum* in 1931 [2]. However, later studies revealed that citrinin can be produced by a variety of fungi, such as *Monascus* [3] and *Penicillium chrysogenum* [4]. A recent study indicated that high amount of citrinin have been found in *Aspergillus niger* fungal species [5]. CTN is a hepato-nephrotoxic mycotoxin commonly occurring in stored grains, beans [6], fruits, juices and vegetables [7]. Research undertaken using a spectroscopy method revealed that *Penicillium expansum* isolated from meat and apples can produce both patulin and citrinin [8]. CTN contamination from major cropping areas in Tunisia had 50% positive wheat samples with an average of 28 μg/kg [9]. CTN contaminants were also reported in tomato juice [10]. The two toxigenic strains of *P. citrinum* and *P. expansum* were able to produce citrinin in cheese at 20 °C, and up to 600 mg of citrinin per kg of cheese was obtained after 10 d of incubation [11]. Many studies have shown that the *Monascus* species produce commercially viable metabolites, including food colorants, cholesterol-lowering agents, and antibiotics [12], and the natural occurrence of citrinin in traditional Chinese food such as red yeast rice has also been investigated [13,14,15]. 

The harmful targets of CTN include the kidney [17], spleen, liver and bone marrow [18], and the cytotoxic effects of citrinin on humans has already been studied. The incubation of embryonic kidney cells (HEK293) with pure CTN at a concentration of 60 μM for 72 h caused 50% of cell death when compared to the control cells [19]. Furthermore, a genotoxicity study of CTN showed a significant concentration-dependent increase in micronucleus (MN) frequency in human lymphocytes [20]. CTN has also been proven to have adverse effects on the reproductive system of adult male mice [21], as well as identified as a teratogenic mycotoxin in female Wistar rats [22]. According to an International Agency for Research on Cancer (IARC) report, the carcinogenicity of CTN has no clear scientific evidence, thus, CTN is classified as a Group 3 carcinogen and its toxicity mechanism remains unknown [23]. This implies that prevention and control of CTN contaminants are very important for safety and security reasons.

The most commonly used analytical methods for CTN detection are thin layer chromatography (TLC) [24], high-performance liquid chromatography (HPLC) [25], liquid chromatography tandem mass spectrometry (LC-MS/MS) [26], ultra-high-performance liquid chromatography and fluorescence detection (UHPLC-FL) [27], gas-chromatography-selected ion monitoring (SIM) mass spectrometry (GC-MS) [28], and an enzyme immunoassay [29]. The advantages of instrument-based methods are their sensitivity and use in simultaneous analysis of multiple mycotoxins; however, there are many disadvantages including the necessity of using complex equipment, incompatibility with real samples, the cost, and amount of time required [30]. The detection of mycotoxins based on monoclonal antibodies is rapid, specific, and sensitive, uses simple equipment, has a low cost, and is compatible with real samples. Furthermore, detection based on monoclonal antibodies through enzyme-linked immunosorbent assay has a low inhibitory concentration at a short period. Indirect competitive ELISA (ic-ELISA) is widely applicable and is an effective assay for the detection of mycotoxins using monoclonal antibodies. Therefore, this study was designed to produce monoclonal antibodies against CTN and to detect the presence of CTN using an indirect competitive enzyme linked immunosorbent (ic-ELISA) assay. 

## 2. Results

### 2.1. Synthesis and Identification of CTN-Protein Conjugates

**Figure 2:**
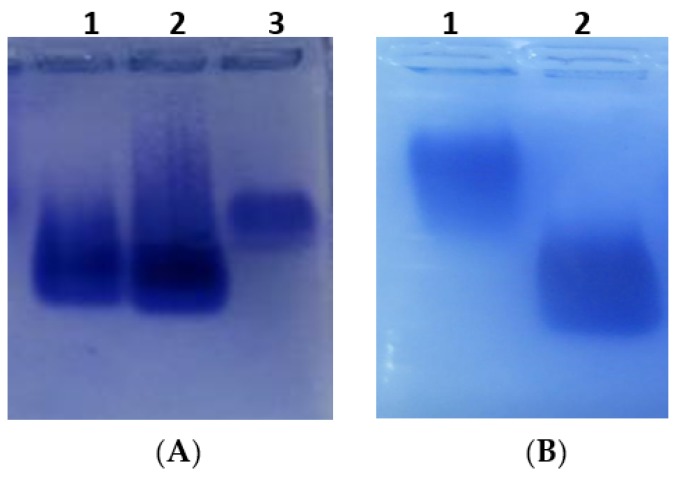
Analysis of the CTN-protein conjugates. (**A**) Non-denaturing agarose gel electrophoresis. Lane 1 and 2, CTN-BSA conjugates. Lane 3, BSA. (**B**) Non-denaturing agarose gel electrophoresis. Lane 1, OVA. Lane 2, CTN-OVA conjugates. BSA: bovine serum albumin; OVA: ovalbumin.

Citrinin is a small non-immunogenic toxin with a molecular weight of 250.25, so it is necessary to conjugate it with carrier proteins for an immuno-response to generate antibodies. In this study, CTN was conjugated to the carrier protein bovine serum albumin (BSA) and ovalbumin (OVA) respectively, through the amide bonds using 1-ethyl-3-(3-dimethylaminopropyl) carbodiimide hydrochloride (EDC) and *N*-hydroxysuccinimide (NHS). Non-denaturing agarose gel electrophoresis was applied to identify the CTN-protein conjugates. After CTN was successfully conjugated with BSA, the CTN-BSA moved faster than that of free BSA (Figure 2A). The non-denaturing agarose gel electrophoresis (Figure 2B) also showed that the migration velocity of the CTN-OVA conjugate was faster than that of free OVA.

### 2.2. Anti-Serum ELISA Assay of the Immunized Mice

A bicinchoninic acid (BCA) assay was carried out to determine the concentration of CTN-protein conjugates, and the results indicated that the concentrations of conjugated CTN-BSA and CTN-OVA were 0.81 and 0.19 mg/mL respectively. Female Balb/C mice were immunized with CTN-BSA conjugates at intervals of 2 weeks. According to the indirect non-competitive ELISA assay, the CTN-BSA immunized mice showed a high anti-serum titer (1:32,000 *v*/*v*) when compared to the non-immunized control mice, indicating that the CTN-BSA conjugates had successfully induced an immuno-response. Therefore, this study used conjugate CTN-BSA for the immuno-antigen, while conjugate CTN-OVA was used as the coating-antigen. 

### 2.3. Cell Fusion and Screening of Hybridoma Cells

The mouse which had a high anti-serum titer was chosen for spleen cell isolation, and the cell fusion was carried out at a ratio of 1:10 B cells from the spleen with Sp2/0 myeloma cells by adding polyethylene glycol (PEG, 1450) drop by drop [31]. Based on our previous cell fusion experience, three fully well grown cell culture dishes of Sp2/0 myeloma cells were fused with spleen cells from one immunized mouse. The hybridoma cells were cultured with aminopterin and a thymidine (HAT) supplementary medium in the presence of feeder cells with 5% CO_2_ at 37 °C and the HAT medium was replaced with hypoxanthine-thymidine (HT) medium 7 d later. Only fused cells grew in HAT medium, and the hybridoma cells increased significantly in number. After growing the fused cells for 10 d, the supernatant titer was determined by indirect non-competitive ELISA using CTN-OVA conjugates as the coating-antigen. Positive clones were transferred to 48-well plates for growth and were subsequently sub-cloned several times until a single positive clone was obtained. Finally, six positive hybridoma cells of interest were screened out and named as 7F2, 5C5, 6B5, 10D3, 21H27 and 12D16 respectively. Cell fusions were successfully performed with an average fusion rate of 99.56% and an average positive rate of 4.5%. The hybridoma cell line 21H27, which stably secreted monoclonal antibodies against CTN, was chosen for further research.

### 2.4. Isotyping and Chromosome Analysis of Anti-CTN mcAb

**Figure 3:**
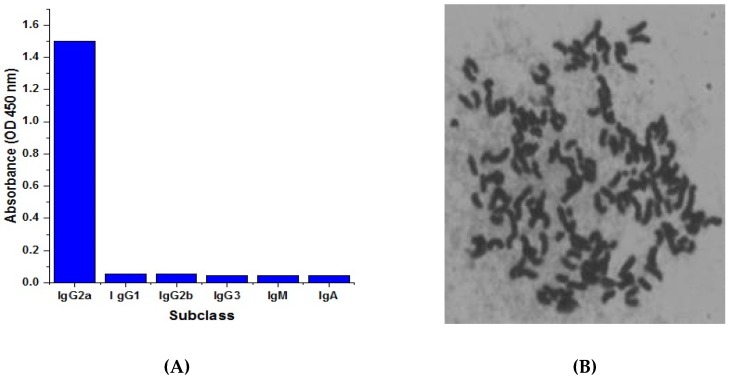
Isotyping and chromosome analysis of anti-CTN mcAb. (**A**) Isotyping of 21H27 cell stably secreting anti-CTN mcAb by using an isotyping kit. (**B**) Chromosome analysis of 21H27 hybridoma cell.

Monoclonal antibodies against CTN were isotyped using a commercial isotyping kit (IgG1, IgG2a, IgG2b, IgG3, IgM, and IgA). From the results seen in Figure 3A, the 21H27 anti-CTN mcAb secreting cell line belonged to the IgG2a subtype. The chromosome numbers of Sp2/0 myeloma cell and spleen cell were 39 ± 1 and 66 ± 4 respectively [32], and the chromosome numbers of hybridoma cell 21H27 were 102 ± 4 (Figure 3B) among the replicates of the experiments. The chromosome number result revealed that positive clone 21H27 was the hybridoma cell produced from the fusion of the spleen cell and the Sp2/0 myeloma cell.

### 2.5. Purification of Anti-CTN mcAb

**Figure 4:**
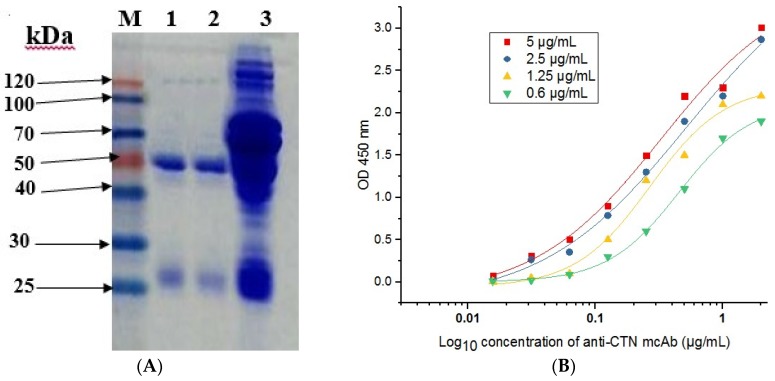
Analysis of purified anti-CTN mcAb. (**A**) Analysis of anti-CTN mcAb purification by sodium dodecyl sulfate-polyacrylamide gel electrophoresis (SDS-PAGE). Lane M, standard protein marker. Lane 1 and 2, purified anti-CTN mcAb. Lane 3, unpurified ascites fluid. (**B**) Affinity result of anti-CTN mcAb at different concentration of coating antigen.

The positive hybridoma 21H27 cell line, which stably secreted anti-CTN mcAb was injected into the pristane pre-treated mice abdomens, and the ascites containing monoclonal antibodies against CTN were produced in the mice abdomen after 8 d of injections. The ascites were withdrawn with a syringe and the monoclonal antibody was purified using the caprylic/ammonium sulfate purification method. Sodium dodecyl sulfate-polyacrylamide gel electrophoresis (SDS-PAGE) was used to assay the antibody, and the result showed that the heavy chain was at 50 kDa and the light chain was at 25 kDa (Figure 4A), indicating that the target antibody was successfully purified.

### 2.6. Affinity and Specificity Test of Anti-CTN mcAb

The affinity of the monoclonal antibody against CTN was determined based on ELISA at different concentrations (5, 2.5, 1.25, and 0.625 μg/mL) of a CTN-OVA coating-antigen. The affinity constant of anti-CTN mcAb was calculated using Microcal Originpro 8.1 data analysis software. The monoclonal antibody secreted by 21H27 positive hybridoma was sensitive to CTN, and the average affinity constant (*Kaff*) of anti-CTN mcAb was 2.0 × 10^8^ L/mol (Figure 4B). To determine the cross-reactivity of this anti-CTN mcAb, competitive ELISA was carried out with structurally related mycitoxins and the molecules Zearalenone (ZEN), Trichothecene (T-2), Patulin (PT), Ochratoxin A (OTA), bovine serum albumin (BSA), ovalbumin (OVA) and Sterigmatocystin (STG). The result indicated that this anti-CTN mcAb was specific to CTN, with no cross reactivity to other molecules and toxins (Table 1).

### 2.7. Standard Curve and Recovery Test

**Figure 5:**
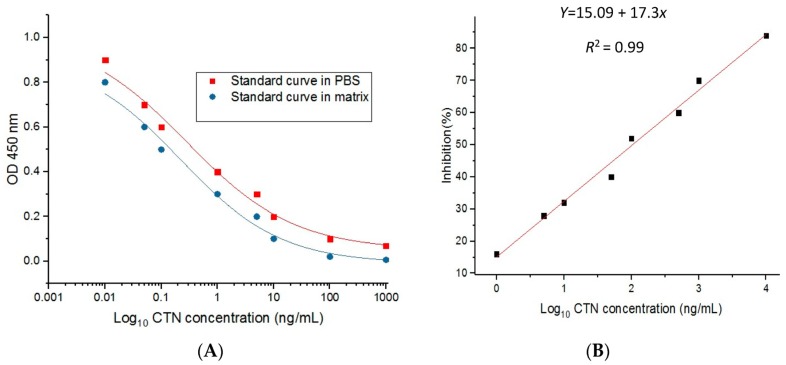
Determination of CTN by ic-ELISA standard curve. (**A**) Standard curve of inhibition competitive ELISA in PBS and in the matrix. The logistic equation was *y* = 1.01613/(1 + (*x*/0.28)0.45497), with the correlation coefficient (*R*^2^) = 0.99. (**B**) Standard linear curve of inhibition competitive ELISA, and the linear equation was *y* = 15.09 + 17*x* with the correlation coefficient (*R*^2^) = 0.99.

Competitive inhibition ELISA was performed using 21H27 anti-CTN mcAb. A standard curve was plotted and the relationship between CTN concentration and its inhibition was analyzed using Microcal Originpro 8.1 data analysis software. The equation of the logistic curve was *y* = 1.01613/(1 + (*x*/0.28)0.45497), and the correlation coefficient (*R*^2^) was about 0.99 (Figure 5A). The linear equation was *y* = 15.09 + 17*x* with the correlation coefficient (*R*^2^) 0.99 (Figure 5B). In this study, the half inhibitory concentration (IC_50_) was 0.28 ng/mL, and the linear range of detection was 0.01–5.96 ng/mL with a lower detection limit (LOD) of 0.01 ng/mL.

In addition, the interference of the matrix was assessed using ic-ELISA at different concentrations of CTN. CTN is commonly found in red yeast rice, wheat feed and food contaminants. Artificially CTN contaminated red yeast rice and wheat were used for the evaluation of the matrix effect. Based on the result, ic-ELISA standard curves were developed in PBS when compared with the matrix (Figure 5A). The matrix interference was reduced by 50-fold dilution, which means that it was appropriate for the ic-ELISA assay. 

### 2.8. Samples Detection by ic-ELISA using Anti-CTN mcAb

To evaluate the precision and accuracy of the developed ic-ELISA, a recovery test was carried out. Non-contaminated red yeast rice was purchased from the supermarket and spiked with CTN at different concentrations (0.5, 5, 100, 1000 ng/mL) (Table 2). The results showed that the recovery range was from 90.2 ± 0.7% to 96.7 ± 1.6% with an average recovery of 93.8 ± 1.6%, and its coefficient of variation (CV) ranged from 1.0% to 4.3% with an average CV of 2.3% (Table 2). Real samples (cheese, wheat bread, apple and tomato) were purchased randomly from the local market to carry out the sample tests. The extracted samples were diluted appropriately to minimize matrix interference and tested by ic-ELISA. The results showed that no CTN toxin was detected in these real samples (Table 3).

## 3. Discussion

Citrinin (CTN) is known as a nephrotoxic mycotoxin, and can also cause cytotoxicity, teratogenicity, hepatotoxicity, and skin irritations in terms of human and animal health [33,34]. CTN is a hapten with a very small molecular weight that cannot induce an immuno-response to produce a monoclonal antibody in mice. Thus, it is necessary to couple CTN with carrier proteins to elicit a specific monoclonal antibody. Structurally, CTN has four active functional groups which can be conjugated with a given carrier protein. Bovine serum albumin (BSA, MW 67,000) has 59 lysine (NH_2_) groups available for coupling [35], and OVA (MW 45,000) has 20 lysine (NH_2_) groups. In this study we applied conjugation of CTN with BSA and OVA, respectively, through the amide bonds using 1-ethyl-3-(3-dimethylaminopropyl) carbodiimide hydrochloride (EDC) and its coupling dehydrating agent *N*-hydroxysuccinimide (NHS). The coupling reaction was carried out in the MES buffer, and the EDC was quenched by 2-mercaptoethanol, making the conjugates stable. Non-denaturing agarose gel electrophoresis results showed that the conjugates of toxin to carrier proteins were successfully obtained. The conjugate result of CTN-BSA was used for immunization and high anti-serum titer was achieved, as was the production of a low IC_50_ in contrast to the previous report of 200 ng/mL IC_50_ [36]. The results of this study showed the successful conjugation of CTN with carrier proteins, suggesting the potential use of these conjugates for immunization and antibody screening. 

The CTN-BSA antigen induced enough anti-serum titers in the Balb/C mice, before the spleen cell from the immunized mice was isolated and fused with Sp2/0 myeloma cells in the presence of polyethylene glycol (PEG, 1450). After screening, a positive hybridoma cell named 21H27 was successfully obtained and injected into the mice abdomens, and the ascitic fluid containing anti-CTN mcAb was purified using a caprylic/ammonium sulfate method. The antibody purification result indicated that the purified target antibody showed significantly clear bands that were different from those of unpurified ascitic fluid (Figure 4A). Our previous study was used as a reference of antibody purification [37]. The antibody secreted by 21H27 hybridoma was specific to CTN, and the affinity constant of anti-CTN mcAb was 2.0 × 10^8^ L/mol. A previous study regarding antibody affinity reported that a certain antibody within 10^7^ to 10^12^ L/mol affinity had good potential for application [38]. Furthermore, this study successfully obtained low LOD with low cross-reactivity to other toxins and molecules under optimum conditions. These results suggested that the antibody secreted by 21H27 was a good antibody and could be used for detection purposes.

Based on the result the ic-ELISA standard curve, the IC_50_ was 0.28 ng/mL and the linear range of detection was 0.01–5.96 ng/mL, which was defined as the concentration of CTN from 20% inhibition to 80%. The lower detection limit (LOD) was 0.01 ng/mL. This indicated that the anti-CTN mcAb secreted by 21H27 could be used to develop an ELISA kit for the detection of CTN. Previous antibody studies regarding CTN were reported with high IC_50,_ and unsatisfactory LOD [36]. Another CTN LOD study was reported, but the conjugation methods were complicated in removing non-conjugated materials by dialysis [39]. The recovery test on a spiked sample showed a 93.8 ± 1.6% mean recovery rate with a 2.3% average coefficient of variation, indicating that the method was appropriate for CTN detection in real samples. Taken together, this anti-CTN mcAb with low LOD, low IC_50_, high affinity and high specificity would provide base information to assess the risk of CTN contamination in feed and foodstuffs and provide insights for further research. 

## 4. Materials and Methods

### 4.1. Materials

Citrinin (CTN), 1-ethyl-3-(3-dimethylaminopropyl) carbodiimide hydrochloride (EDC), *N*-hydroxysuccinimide (NHS), 2-(morpholino) ethanesulfonic acid (MES), 2-mercaptoethanol, methanol, bovine serum albumin (BSA), ovalbumin (OVA), polyethylene glycol, 1450 (PEG 1450), Freund’s complete adjuvant, Freund’s incomplete adjuvant, horseradish peroxidase (HRP)-conjugated goat anti-mouse IgG, hypoxantine, aminopterin and thymidine (HAT) medium, hypoxanthine-thymidine (HT) medium, mouse monoclonal antibody isotyping reagent (IgG1, IgG2a, IgG3, IgM, IgA), and RPMI 1640 were purchased from Sigma-Aldrich Chemical (St. Louis, MO, USA). The Sp2/0 myeloma cell was stored within our laboratory in liquid nitrogen. Female Balb/C mice (8-weeks old) were purchased from the Wushi animal laboratory (Shanghai, China). All other reagents were chemical grade and obtained from commercial sources in China.

### 4.2. Synthesis and Characterization of CTN-Protein Conjugates

CTN was conjugated with the carrier proteins bovine serum albumin (BSA) or ovalbumin (OVA), through the amide bonds using 1-ethyl-3-(3-dimethylaminopropyl) carbodiimide hydrochloride (EDC) and *N*-hydroxysuccinimide (NHS) with slight modifications [40]. CTN (1 mg) was mixed with 1 mL of MES buffered saline (containing 0.1 M MES (2-(morpholino) ethanesulfonic acid), 0.5 M NaCl, pH 6.0). After 0.2 mg of EDC and 0.3 mg of NHS were added to the above reaction mixture, it was incubated for 15 min at room temperature. EDC was then quenched by 1.6 μL 2-mercaptoethanol in the MES buffer. Subsequently, 0.5 mL protein solution (10 mg/mL BSA in 0.1 M phosphate buffered saline (PBS)) was added. The reaction mixture was well mixed and, reacted for 2 h at room temperature. Next, the conjugates were dialyzed with 0.01 M phosphate buffered saline (PBS) at 4 °C for 3 d, where freshly prepared PBS was replaced every 8 h. The CTN-BSA conjugate was obtained and lyophilized at the evaporation rotary machine until dry for about 3 h. The final products were stored at −20 °C for further use. The coating-antigen CTN-OVA was prepared with the same method for CTN-BSA as mentioned above.

### 4.3. Analysis of CTN-Protein Conjugates

CTN-BSA and CTN-OVA conjugates were checked by non-denaturing agarose gel electrophoresis [37] and 20 μL of the conjugates were mixed with 10 μL of protein loading buffer, before the mixture was loaded to 1% agarose gel and run at 200 V for 50 min. After electrophoresis, the gel was stained overnight with coomassie blue R-250 and then destained until the clear bands were seen. CTN has maximal UV absorption at 250 nm and 333 nm (in methanol). CTN conjugation with BSA and OVA carrier proteins was also reported from previous studies [40,41]. A bicinchoninic acid (BCA) assay was used to determine the concentrations of CTN-protein conjugates [37,42].

### 4.4. Mice Immunization and Anti-Serum ELISA Assay

Healthy female Balb/C mice (8-weeks old) were prepared for immunization, and all mice were cared for according to the institutional guidelines of the Fujian Agriculture and Forestry University, China. CTN-BSA conjugates (100 μg/mL) dissolved in PBS were emulsified with an equal volume of Freund’s complete adjuvant before female Balb/C mice at multiple sites were immunized through subcutaneous injection. After a two-week interval, the mice were boosted by 50 μg/mL of CTN-BSA conjugate emulsified with an equal volume of Freund’s incomplete adjuvant. After boosting the mice five times, serum from the mice tail was collected and the titer of anti-serum was tested by indirect ELISA. The coating-antigen CTN-OVA was diluted to 5 μg/mL, then ELISA plates were coated with 100 μL/well coating-antigen and incubated overnight at 4 °C then washed by PBST (PBS with 0.05% of Tween-20) and PBS for three times, respectively. After washing, 200 μL/well of PBSM (PBS with 5% non-fat milk) were added to block residual protein-binding sites for 2 h at 37 °C. After washing three times by PBST and PBS, respectively, 100 μL/well of anti-CTN anti-serum was added and incubated for 1.5 h at 37 °C. After washing further, three times, with PBST and PBS, respectively, 100 μL/well of HRP conjugated goat anti-mouse IgG (1:8000) was added and incubated at 37 °C for 1.5 h. The plates were then washed three times with PBST and PBS, respectively, and 100 μL/well TMB substrate (mixed equal volumes of TMB buffer A and buffer B) were added and incubated for 15 min at 37 °C. The reaction was stopped by the addition of 50 μL/well of H_2_SO_4_ (2 mol/L), and the absorbance was then measured with a 450 nm micro plate reader [37]. 

### 4.5. Cell Fusion and Screening of Anti-CTN mcAb

Production of hybridoma cell against CTN was based on a standard method [37,42,43] with slight modifications. After anti-serum titer was tested, the high titer mouse was chosen and immunized with CTN-BSA without adjuvant through intraperitoneal 3 d before cell fusion. Sp2/0 myeloma cells grew well at a complete medium, and the spleen cells from the immunized mouse were fused at 1 ratio of Sp2/0 with 10 B cells from the spleen cells. The fusion was done by adding polyethylene glycol (PEG, 1450) drop by drop [31], and the hybridoma were cultured with a HAT supplementary medium in the presence of feeder cells in 96-well plates with 5% CO_2_ at 37 °C. One week later, the HAT medium was changed to a HT medium. After a further 10 d incubation, the presence of anti-CTN antibodies was checked from the supernatant of the hybridoma cells using ELISA. The positive clones were transferred to 48-well plates and subsequently sub-cloned several times through limited dilution method.

### 4.6. Isotyping and Chromosome Analysis of the Anti-CTN mcAb

After hybridoma of interest were subcloned, the isotyping of the positive cells was conducted according to the guide with minor modifications [37]. For chromosome analysis, geimsa staining was applied [44] and the chromosome was observed under a microscope [37,42]. 

### 4.7. Production and Purification of Anti-CTN mcAb

Healthy mature female Balb/C mice were injected intraperitoneally with 500 μL of pristane (2, 6, 10, 14-Tetramethylpentadecane) 7 d before receiving ip injection of the hybridoma cell line 21H27, and ascites fluid was developed after injection for 8 d. The ascites fluid was harvested from the mice and centrifuged at 9391× *g* for 10 min, and the antibody was purified using the caprylic/ammonium sulfate precipitation method [45]. The purified mcAb was analyzed by SDS-PAGE [46], and a BCA protein assay kit was used to determine the concentration of the ascites fluid and the purified mcAb [42].

### 4.8. Affinity Assay and Cross-Reactivity of Anti-CTN mcAb

An affinity assay for monoclonal antibodies against CTN was carried out according to previous publications in our laboratory [37,42] with minor modifications. The coating antigen (CTN-OVA) was coated at different concentrations (5, 2.5, 1.25, 0.625 μg/mL) overnight at 4 °C, then washed with PBST and PBS three times, respectively. PBSM (200 μL/well) was added to block residual protein-binding sites, and incubated for 2 h at 37 °C. After washing, anti-CTN mcAb was serially diluted, and 100 μL/well of anti-CTN mcAb was added and incubated for 1.5 h at 37 °C. After washing, 100 μL/well HRP conjugated goat anti-mouse IgG (1:8000) was added and incubated at 37 °C for 1.5 h. After washing three times with PBST and PBS, respectively, 100 μL/well of TMB substrate was added for color development, and incubated at 37 °C for 15 min. The reaction was stopped by adding 50 μL/well of H_2_SO_4_ (2 mol/L). The absorbance was measured at 450 nm by the micro plate reader. Data were analyzed using Microcal OriginPro 8.1 data analysis software, and the affinity constants of anti-CTN mcAb were calculated according to the standard method described in Reference [47]. The specificity and cross-reactivity of anti-CTN mcAb were carried out according to references from our previous study [37]. Structurally related molecules and toxins zearalenone (ZEN), trichothecene (T-2 patulin (PT), ochratoxin A (OTA), bovine serum albumin (BSA), ovalbumin (OVA), and sterigmatocystin (STG) were used as competitor antigens of CTN, and cross-reactivity was calculated as: cross-reactivity (%) = concentration of standard CTN inhibiting 50% of antibody binding divided by the concentration of competitor inhibiting 50% of antibody binding multiplied by 100%.

### 4.9. Standard Curve and Recovery Test

Artificially contaminated samples with CTN were tested using competitive indirect ELISA. The CTN-OVA coating antigen was prepared at an optimum concentration. The ELISA micro wells were coated overnight at 4 °C. After washing with PBST and followed by PBS for three times, the wells were blocked with 200 μL/well of 5% PBSM then incubated for 2 h at 37 °C. After washing, an equal volume of anti-CTN mcAb was reacted with a free CTN toxin at different concentrations (0, 0.01, 0.05, 0.1, 1, 5, 10, 100, 1000 ng/mL). The reaction products were incubated for 30 min at 37 °C. Subsequently, 100 μL/well of the product was transferred to ELISA micro wells with three replicates of each concentration standard, and incubated for 1 h at 37 °C. After washing, HRP conjugated goat anti-mouse IgG (1:8000) was diluted in 5% PBSM and 100 μL/well was added. After incubating and washing, 100 μL/well TMB substrate solution was added and incubated for 15 min at 37 °C. The reaction was stopped by adding 50 μL/well of H_2_SO_4_ and absorbance was measured at 450 nm. The linear range to detect CTN was defined as the concentration of CTN towards from 20% to 80% inhibition [48]. The inhibition concentration of CTN toxin in relation to anti-CTN mcAb was analyzed using Microcal OriginPro 8.1 [37,42]. 

Red yeast rice (1 g) and wheat flour samples were spiked with the CTN toxin at different concentrations (0.5, 5, 100, 1000 ng/mL). Added to each sample was 10 mL of 70% ethanol (*v*/*v*) and mixed well by ultrasonic treatment for 30 min. The sample was then extracted at room temperature. After being centrifuged at 1055× *g* for 20 min, the supernatant was used for ic-ELISA assay. The matrix effect in the standard curve was tested by comparing the sample in the matrix and in the PBS. The sample extract was diluted to reduce matrix interference. Recovery tests were performed and determined based on the standard curve. The coefficient variation and the recovery tests were determined from data triplicates [42].

### 4.10. Ethical Statement and Animal Care 

All animal experiments were performed according to the Animal Ethics Committee of the Fujian Agriculture and Forestry University in China (C1017/23.12.2014). All female Balb/C mice were kept in a well-designed mice room with three mice per cage. The temperature of the room was set at 24 ± 2 °C with an approximate relative humidity of 50%–60%. The mice were given clean water and fed with Forti-Diet commercial pellet mouse food. Overall health monitoring of the mice were performed regularly and the mice room was cleaned on a regular basis. 

## Figures and Tables

**Table 1:** Cross-reactivity of anti-CTN mcAb with structurally related toxins and molecules.

Toxins	Cross-Reactivity (%)
ZEA	<0.01%
PT	0.01%
STG	<0.01%
T-2	<0.01%
OTA	<0.01%
OVA	<0.01%
BSA	<0.01%

**Table 2:** Recovery test of CTN spiked red yeast rice (*n* = 3).

Spiked Level (ng/mL)	Measured Concentration (ng/mL)	Recovery (%)	CV (%)
0.5	0.47 ± 0.01	94.5 ± 1.1	1.5
5	4.84 ± 0.06	96.7 ± 1.6	2.2
100	93.9 ± 3.0	93.9 ± 3.0	4.3
1000	902.0 ± 7.2	90.2 ± 0.7	1.0
Average		93.8 ± 1.6	2.3

Note: ± indicates value of the average deviation from the mean. Data were given as the mean value. The coefficient of variation (CV) was defined as the ratio of the standard deviation to the mean in the recovery test.

**Table 3:** Analysis of CTN toxin in the real sample.

Samples	OD 450 nm	Detection Results
Control PBS (B0)	1.07 ± 0.11	ND
Wheat bread	1.1 ± 0.07	ND
Cheese	1.03 ± 0.04	ND
Apple	1.00 ± 0.04	ND
Tomato	1.13 ± 0.09	ND

Note: ND means no CTN was detected out in the above samples tests. ± indicates the value of the average deviation from the mean. Data were given as the mean value.
